# Efficient, Stable, and Low-Cost PbS Quantum Dot Solar Cells with Cr–Ag Electrodes

**DOI:** 10.3390/nano9091205

**Published:** 2019-08-27

**Authors:** Jobeda J. Khanam, Simon Y. Foo, Zhibin Yu, Tianhan Liu, Pengsu Mao

**Affiliations:** 1 Department of Electrical and Computer Engineering, FAMU-FSU College of Engineering, Tallahassee, FL 32310, USA; 2 Department of Industrial and Manufacturing Engineering, High-Performance Materials Institute, FAMU-FSU College of Engineering, Florida State University, Tallahassee, FL 32310, USA; 3 Department of Physics, Florida State University, Tallahassee, FL 32306, USA

**Keywords:** PbS quantum dot, solar cell, deposition method, PbS concentration, storage stability

## Abstract

PbS quantum dots (QDs) are a promising nanostructured material for solar cells. However, limited works have been done to explore the active layer thickness, layer deposition techniques, stability improvement, and cost reduction for PbS QD solar cells. We address those issues of device fabrication herein and suggest their possible solutions. In our work, to get the maximum current density from a PbS QD solar cell, we estimated the optimized active layer thickness using Matlab simulation. After that, we fabricated a high-performance and low-cost QD photovoltaic (PV) device with the simulated optimized active layer thickness. We implemented this low-cost device using a 10 mg/mL PbS concentration. Here, spin coating and drop-cast layer deposition methods were used and compared. We found that the device prepared by the spin coating method was more efficient than that by the drop cast method. The spin-coated PbS QD solar cell provided 6.5% power conversion efficiency (PCE) for the AM1.5 light spectrum. Besides this, we observed that Cr (chromium) interfaced with the Ag (Cr–Ag) electrode can provide a highly air-stable electrode.

## 1. Introduction

The semiconductor quantum dots (QDs) of groups IV and VI make compounds PbSe and PbS. These semiconductors are commonly known as photo-absorbers in the near-infrared and visible regions of the light spectrum. PbS QDs have achieved recognition for the generation of multiple excitons, huge bandgap tunability, and comparatively easy solution methods [1,2,3,4,5,6]. Colloidal QDs (CQDs) are generally inorganic semiconductor materials with organic molecules on their surface. By using surface treatment, those materials can perform like either a positive (p-type) or negative (n-type) semiconductor. This property enables their widespread usage in the architecture of optoelectronic organic, inorganic, and hybrid devices [7]. PbS QD material has been used and studied recently in applications in bilayer photodetectors [8,9], solar cells [10,11,12], cell imaging [13], and light-emitting diodes [14]. The photovoltaic device architecture and the QD surface ligands play important roles in determining the optoelectronic properties of QD solar cells [15,16]. CQD materials consist of individual QDs where the QDs remain side by side [17,18].

The surface morphology plays a vital role in achieving an efficient PbS QD solar cell. Because of the quantum dots, the surface-area-to-volume ratio becomes higher in QD materials, which causes electronic traps. Those traps raise the chance of charge recombination [19]. For continuous charge transfer and separation, the solar cell surface morphology needs to be considered and improved. The film quality depends on the quantum dot size [20], the concentration of PbS QD material, ligand exchanger and exchanging time [15], an optimal annealing process [21], band alignment [22], and the solvent’s properties. The rate of evaporation, viscosity, and dispersibility are vital properties of the solvent; these properties help to obtain pinhole-free and crack-free surfaces [23]. Changing the ligand exchange time can also provide excellent surface quality. Proper bandgap alignment of the device material prevents charge recombination, hence reducing series resistance. Therefore, choosing the optimum solvent, PbS QD concentration, annealing temperature, and ligand exchange time can provide excellent film properties.

Extensive research on PbS QD solar cells has been conducted via solvent engineering and bandgap alignment [22,23]. However, few works have been performed on photoactive layer thickness estimation, stability improvement, cost reduction, and the layer deposition process. In optoelectronic device fabrication, slot-die coating and screen printing [24] proved to be a product-compatible method for microfilm deposition. For thin-film (nano range) photovoltaic devices, these methods are not applicable. A blade coating [25] deposition method is generally used for thin film deposition, but PbS QD solution is not viscous enough [26] to implement the blade coating process.

There are some critical issues in PbS QD photovoltaic (PV) device fabrication. We focus herein on four issues in PbS QD solar cells. Firstly, the active layer thickness for fabricating the device is unknown. For any device fabricated without knowing the thickness of PbS QDs, we might not achieve the maximum current density. Secondly, for layer deposition, the spin coating method was used in most previous work [27,28]. The other layer disposition methods for PbS QD solar cells have not been sufficiently explored. The third issue is the high cost of PbS QD solar cells. Most research works suggest using PbS QDs at a high concentration (40 mg mL^−1^ to 100 mg mL^−1^) to make a working device [29,30]. This high concentration of PbS QDs increases the device price as much material is wasted during spin coating. Finally, using only Ag material as a back electrode does not provide device stability, because Ag oxidizes quickly and forms an Ag_2_O intermediate layer [31,32].

It is essential to design a low-cost and more stable CQD solar cell to solve these four issues. Here, in our work, we firstly created a Matlab simulation for a PbS QD photovoltaic device [33]. We estimated the optimized thickness to get the maximum current density. Then, we fabricated an inverted-structure ZnO/PbS QD device and aimed to attain the simulated optimized active layer (PbS QD) thickness. To explore the deposition method, we fabricated and compared the performance of PbS QD solar cells using drop cast and spin coating methodologies. For cost optimization, we used 10 mg mL^−1^ PbS QDs instead of highly concentrated PbS QDs. To improve the air stability performance of the back electrode, we used a Cr–Ag electrode because the Cr layer provides excellent stability to silver and sticks well to the surface of the cell. Thin layers of Cr (5–10 nm) do not usually modify the properties of the devices [34,35].

## 2. Materials and Methods

### 2.1. Materials

We obtained ITO (Indium Tin Oxide) coated glass substrates (110 nm, 8–12 Ω/sq), PbS core-type quantum dots, 1,2-ethanedithiol (EDT), tetrabutylammonium iodide (TBAI) and all solvents from Sigma-Aldrich (St. Louis, MO, USA). We used those materials without additional refinement or alteration. Cr (99.9%) and Ag (99.9%) pellets were obtained from Lesker (Jefferson Hills, PA, USA).

### 2.2. Estimation of the PbS QD Layer Thickness 

**Figure 1:**
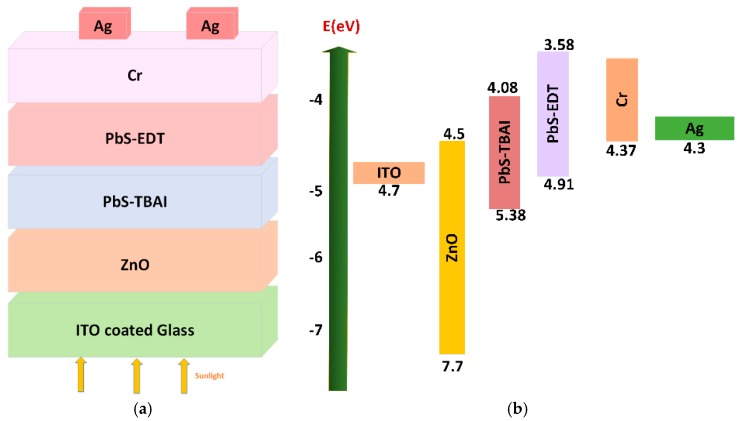
(**a**) Schematic diagram and (**b**) energy level diagram of the PbS quantum dot (QD) photovoltaic device used in this study.

The structure of our PbS QD solar cell was ITO/ZnO/PbS-TBAI/PbS-EDT/Cr/Ag. A schematic and band diagram for our device are shown in Figure 1a,b.

**Figure 2:**
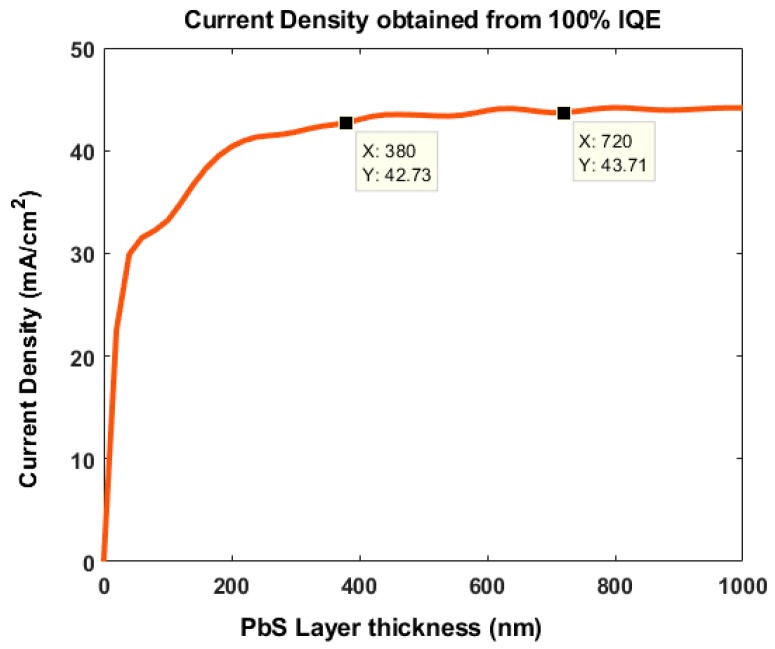
Variation of the active layer thickness to achieve the maximum current density.

We estimated the thickness of the PbS QD layer using the process reported by Khanam et al. [33]. The Matlab simulation processed the same device structure shown in Figure 1a. By varying the thickness of the PbS, we found that a PbS thickness of ≥380 nm provided the maximum current density of 43 mA/cm^2^ (Figure 2). The current density obtained from 100% Internal Quantum Efficiency (IQE).

### 2.3. Device Fabrication

On the ITO coated glass substrates, we fabricated our device (ITO/ZnO/PbS-TBAI/PbS-EDT/Cr/Ag). We used 1 M HCL in the etching process of the ITO coated glass to avoid short circuiting. We firstly used detergent to clean the substrate. Deionized water, isopropanol, and acetone were subsequently used to clean the substrate. We dried the substrates in a vacuum oven and performed oxygen plasma treatment for about 5 min. ZnO nanoparticle solution was spin coated at 2000 rpm on the substrate for approximately 20 s to get an 80 nm thick ZnO layer. Then, we heated the ITO/ZnO substrate for 20 min at 110 °C. For both spin coating and drop cast device fabrication, oleic-acid-coated PbS QDs with a concentration of 10 mg/mL in toluene solvent were used. For the ligand exchange process, 1,2-ethanedithiol (EDT) and tetrabutylammonium iodide (TBAI) were used. As organic and inorganic ligands, EDT solution (0.04 vol % in acetonitrile (ACN)) and TBAI solution (10 mg mL^−1^ in methanol) were used, respectively. Due to ligand interchange, the device lost surface layer volume. This generated cracks on the surface. To remove those cracks, we used ACN as a rinsing solvent. We implemented the spin coating and drop cast layer deposition methods in open air and at ambient temperature.

#### 2.3.1. The Drop Cast Deposition Method for Device Fabrication

Active layers were deposited on the ITO/ZnO substrate using layer-by-layer (LbL) deposition in the drop cast method. We built two-, three-, four-, five-, and six-layered devices using the drop cast method to determine which layer number produced the maximum efficiency. For the PbS-TBAI photoactive layer deposition, ~30 μL of PbS QDs was dropped and allowed to dry completely on the ITO/ZnO substrate. Then, a TBAI solution was dropped onto the substrate and left for 40 s. We rinsed the substrate two times using ACN at 2000 rpm. For the PbS-EDT photoactive layer deposition, ~30 μL of PbS QDs was dropped and allowed to dry completely on the ITO/ZnO substrate. After that, EDT solution was dropped onto the substrate and left for 40 s. We used the same rinsing process as above. Finally, we heated the device at 110 °C for 5 min. The layered substrate was preserved in open air overnight. After that, for electrode evaporation, we moved the substrate to a nitrogen (N_2_)-filled glove box. For thermal evaporation, the device was covered on the edges with Kapton tape and mounted on a sample holder with carbon tape. The chamber was pumped down to 5 × 10^−7^ torr base pressure before evaporation. Then, 5 nm Cr and 100 nm Ag were thermally evaporated at rates of 0.7 Å s^−1^ and 1 Å s^−1^, respectively, at reduced pressure (<10^−6^ Torr). During thermal evaporation, we used a shadow mask. The photoactive area of our device was 1 mm^2^.

#### 2.3.2. The Spin Coating Deposition Method for Device Fabrication

In the spin coating method, the photoactive layers were fabricated on the ITO/ZnO substrate by LBL deposition. We built a seven-layered device with this method. For the PbS-TBAI photoactive layers on the device, ~30 μL of PbS QDs was dropped onto the ITO/ZnO substrate for 90 s so as to adhere well to the glass. Then, the substrate was spin coated for 10 s at 2500 rpm. Then, TBAI solution was dropped onto the substrate and left for 40 s. We subsequently rinsed the substrate two times using ACN at 2000 rpm. For the PbS-EDT photoactive layer deposition, ~30 μL of PbS QDs was dropped and left for 90 s on the ITO/ZnO substrate. Then, the substrate was spin coated for 15 s at 2500 rpm. After that, EDT solution was dropped onto the substrate and left for 40 s. We used the same rinsing process as above. After deposition of two to three photoactive layers, we heated the substrate at 80 °C for 5 min. Then, we deposited the Cr–Ag electrode by using thermal evaporation. The active device area was about 3 mm^2^.

### 2.4. Device Characterization and Instrumentation

It is required to know the current density–voltage (J–V) characteristics of a device to measure its efficiency. Here we used a Keithley 2400 instrument at light intensity 100 mW/cm^2^ to measure the J–V characteristics. We investigated the surface structure and a cross-sectional view using a field emission scanning electron microscope (FESEM). We used a UV–vis–NIR spectrophotometer (PerkinElmer Co, Waltham, MA, USA) to obtain the absorption spectra. The optical images were captured using an Olympus BX40 microscope with a 5× lens and a CCD camera (Teledyne Photometrics Co, Tucson, AZ, USA)

## 3. Results and Discussion

**Figure 3:**
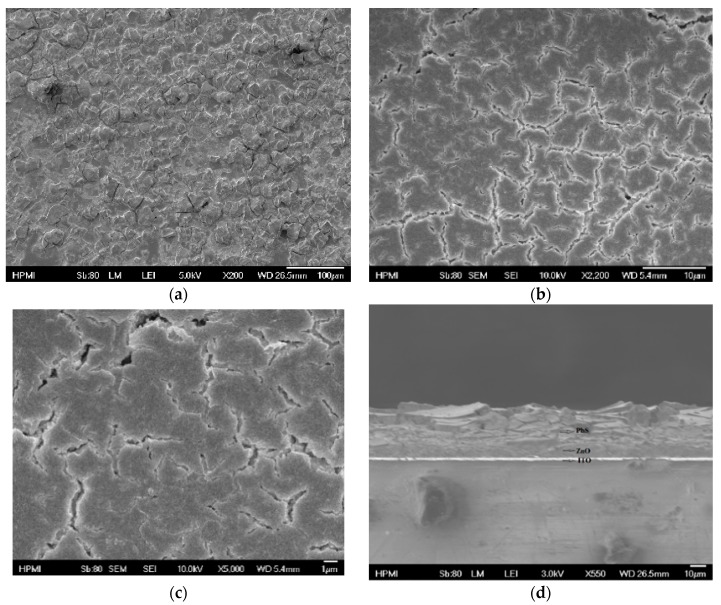
Field emission scanning electron microscope (FESEM) images of TBAI and EDT exchanged film with different magnifications: (**a**) 100 μm; (**b**) 10 μm; (**c**) 1 μm. (**d**) Cross-sectional image of the sample made using the drop cast method.

**Figure 4:**
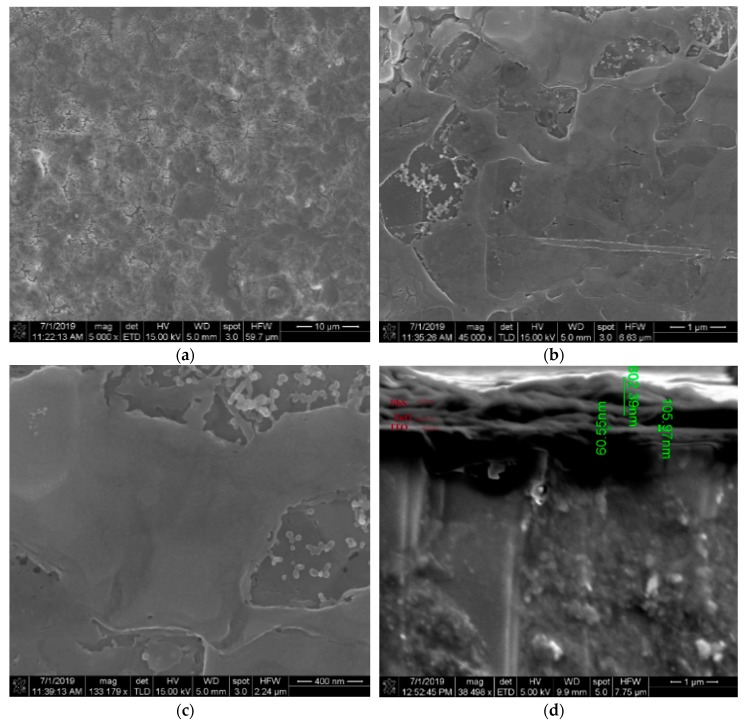
FESEM images of seven-layered TBAI and EDT exchanged film made using the spin coating method with different magnifications: (**a**) 10 μm; (**b**) 1 μm; (**c**) 400 nm. (**d**) Cross-sectional image of the sample made using the spin coating method.

We characterized and analyzed our fabricated ITO/ZnO/PbS-TBAI/PbS-EDT/Cr/Ag inverted-structure solar cells. We observed the surface film morphology of both the drop cast and spin-coated PbS QDs using FESEM with different magnifications. For the drop cast deposition method, we observed several cracks on the film surface of the device, as shown in Figure 3a–c. However, for the spin coating deposition method, we observed that the device surface contained almost no cracks (Figure 4a–c). In our spin-coated device, the film surface, although crack free, was not uniform. To get a uniform surface, we may need to anneal the device after each active layer deposition. Also, making the PbS QD materials adhesive using a viscous solvent might improve the surface quality.

A cross-sectional view was required to explore the actual device and estimate the thickness of the layers. In Figure 3d, we can see large cracks and nonuniformity in the cross-sectional view of the drop-cast device. In Figure 4d, we can see that for the spin-coated device, the film thickness was 802 nm, and the cross section was crack free.

**Figure 5:**
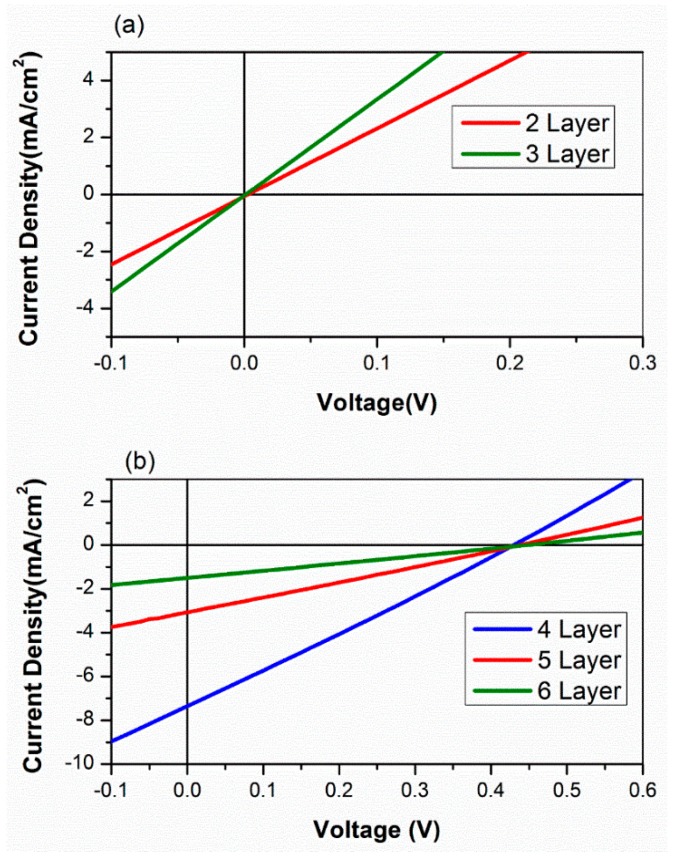
The J–V characteristics of PbS QD solar cells with different numbers of photoactive layers prepared using a drop cast method: (**a**) two or three layers; (**b**) four, five, or six layers.

The J–V characteristics of the photovoltaic devices for different layers made using the drop cast method are shown in Figure 5a,b. We can see in Table 1 that the drop-cast devices consisting of two, three, four, five, and six layers of PbS showed power conversion efficiency (PCE) values of 0%, 0%, 1.5%, 0.55%, and 0.2%, respectively. We also observed that the open-circuit voltage (V_oc_) was 0.4 V, but the current density (J_sc_) varied in the drop-cast working devices (with four, five, and six layers). The four-layered PbS device showed a 1.5% PCE. The results show that the drop cast method can provide a working device but with very low efficiency.

**Figure 6:**
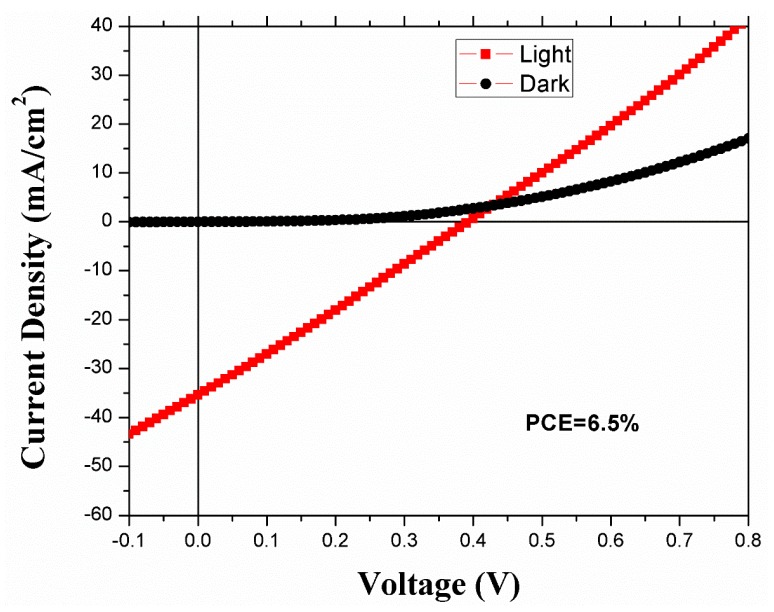
The J–V characteristics of the seven-layered spin-coated device.

The J–V characteristics of the photovoltaic devices made using the spin coating method are shown in Figure 6. The device consisting of seven layers of PbS showed a PCE of 6.5%. The previously reported PCE was 6.0%, where the device (FTO/TiO_2_/PbS) used an Au/Ag anode [16,22]. Hence, our device shows an improvement with the Cr/Ag electrode. From Table 1, we can see that we achieved a V_oc_ of ~0.38 V, current density (J_sc_) of ~35 mA/cm^2^, and FF of ~0.5 in the spin-coated device.

For both the spin-coated and the drop-cast device, we observed low FF values. For PbS QD solar cell fabrication, most papers suggest using an open-air and ambient-temperature process. We noticed that the concentration did not play a crucial role in the process. Previous studies used highly concentrated PbS QDs (30–50 mg/mL) but got low fill factors (40–50%) [1,2,6,8,29]. Humidity control is required for the fabrication process of the photovoltaic device [36]. Photovoltaic devices with organic molecules are highly sensitive to oxygen and moisture. They degrade due to undesired oxidation and hydrolysis [33]. N_2_ lessens the concentration of oxygen and reduces the oxidation effect [37]. Therefore, to make efficient devices, we need to use optimal conditions. This can be achieved by using an N_2_-filled glove box with controlled relative humidity during the fabrication of the device.

**Figure 7:**
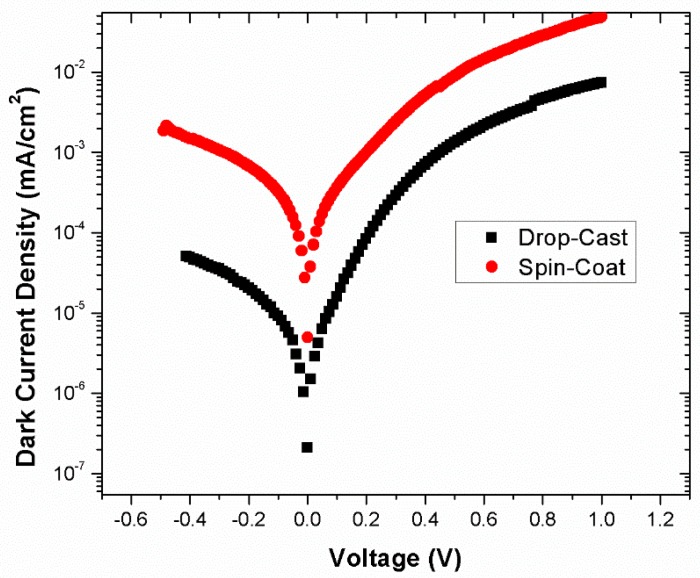
Dark J–V graph in log scale for drop-cast (four-layered) and spin-coated devices.

The dark J–V graphs in log scale for both the drop-cast (four-layered) and spin-coated working devices are shown in Figure 7. This figure shows that the leakage current was much lower for both the spin-coated and drop-cast devices. In the forwarding direction, both devices showed a very small current density, which represents a higher rectification ratio. Hence, both devices had excellent dark J–V characteristics.

**Figure 8:**
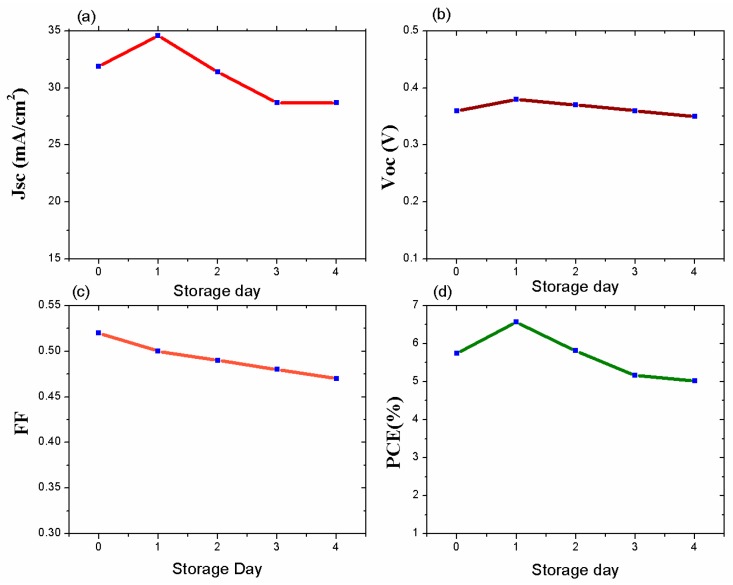
The evolution of the photoactivity parameters with the number of storage days: (**a**) J_sc_; (**b**) V_oc_; (**c**) FF; (**d**) PCE.

We conducted a stability test on the spin-coated device. The device showed air exposure stability over five days without any encapsulation (Figure 8). We preserved the device in an N_2_-filled glovebox, and during stability testing, we exposed the device to open air. From Figure 8, we can see that on the first day, the PCE became high; however, on the remaining days, the PCE was stable. This PCE improvement is questionable as further PbS QD oxidation took place the following day. The cause of this initial performance hike due to short-term air exposure is still under investigation.

**Figure 9:**
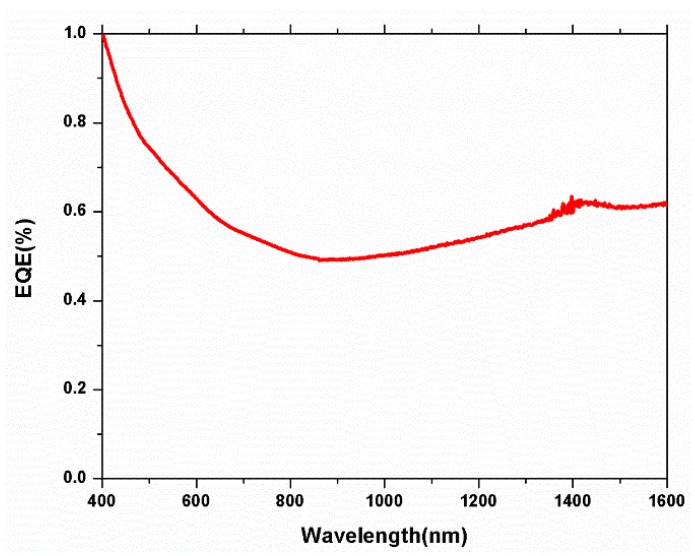
The external quantum efficiency (EQE) of our fabricated spin-coated device.

The Matlab simulation results on the external quantum efficiency (EQE) of our device are shown in Figure S1 (Supporting Information). We can see from Figure S1 that the device absorbed the light spectrum from 400 nm to 1600 nm. The EQE of our fabricated device also showed a similar outcome (Figure 9).

To investigate the necessity of LbL spin coating deposition, we took optical images of the one- and five-layer spin-coated film surfaces. The one-layer spin-coated PbS surface showed many pinholes (Figure S2a, Supporting Information), whereas the five-layer spin coating showed no pinholes on the surface (Figure S2b, Supporting Information).

We analyzed the stability of back electrodes containing a Cr–Ag layer and those of solely Ag during exposure to air. We took optical images of the devices after five days of air exposure. We observed that the surface without Cr became cracked (Figure S3a, Supporting Information), whereas the Cr–Ag electrode was stable and uniform (Figure S3b, Supporting Information).

## 4. Conclusions

In summary, we developed low-cost and stable PbS QD solar cells. We addressed some issues in device fabrication and explored their possible solutions. We built quantum dot solar cells by exploring two types of deposition method while using a Cr–Ag electrode. All our devices were fabricated in ambient temperatures. The device cost was reduced as we used 10 mg/mL PbS QDs instead of highly concentrated PbS QDs. Our experimental results show that the drop cast method can provide a working device, but the efficiency was quite a lot lower than that of the spin-coated device. Moreover, the spin coating method provides a uniform layer, while the drop casting method makes many cracks in the surface. Hence, we found that spin coating is the best solution for layer deposition during device fabrication. We found that the Cr–Ag interfacial layer provided air stability in the electrode surface.

## Figures and Tables

**Table 1:** Device parameters obtained from spin coating and drop cast methods.

Deposition Process	Layer Deposition	V_oc_ (V)	J_sc_ (mA/cm^2^)	Fill Factor (FF) (%)	PCE (%)
Spin coating	5 PbS-TBAI + 2 PbS-EDT (7 Layers)	0.38	35	50	6.5
Drop cast	1 PbS-TBAI + 1 PbS-EDT (2 layers)	0	0	0	0
	2 PbS-TBAI + 1 PbS-EDT (3 layers)	0	0	0	0
	2 PbS-TBAI + 2 PbS-EDT (4 layers)	0.4	7.5	50	1.5
	3 PbS-TBAI + 2 PbS-EDT (5 layers)	0.4	3	46	0.55
	4 PbS-TBAI + 2 PbS-EDT (6 Layers)	0.4	1	48	0.2

## Supplementary Materials

The following are available online at https://www.mdpi.com/2079-4991/9/9/1205/s1, Figure S1: EQE spectra for the simulated results for our device, Figure S2: a. One layer of spin-coated film surface has many pinholes b. Four layers of spin-coated film surface has no pinhole, Figure S3: a. Ag electrode causes crack on the surface b. Cr-Ag electrode causes no crack on surface after five days.
