# Supplementary material for: Efficient, Stable, and Low-Cost PbS Quantum Dot Solar Cells with Cr–Ag Electrodes
Source: Nanomaterials (Basel). 2019 Aug 27;9(9):1205. doi: 10.3390/nano9091205 (PMC6780186; doi:10.3390/nano9091205)
Supplement: Supplementary file 1 [file nanomaterials-09-01205-s001.pdf]

Supplementary material

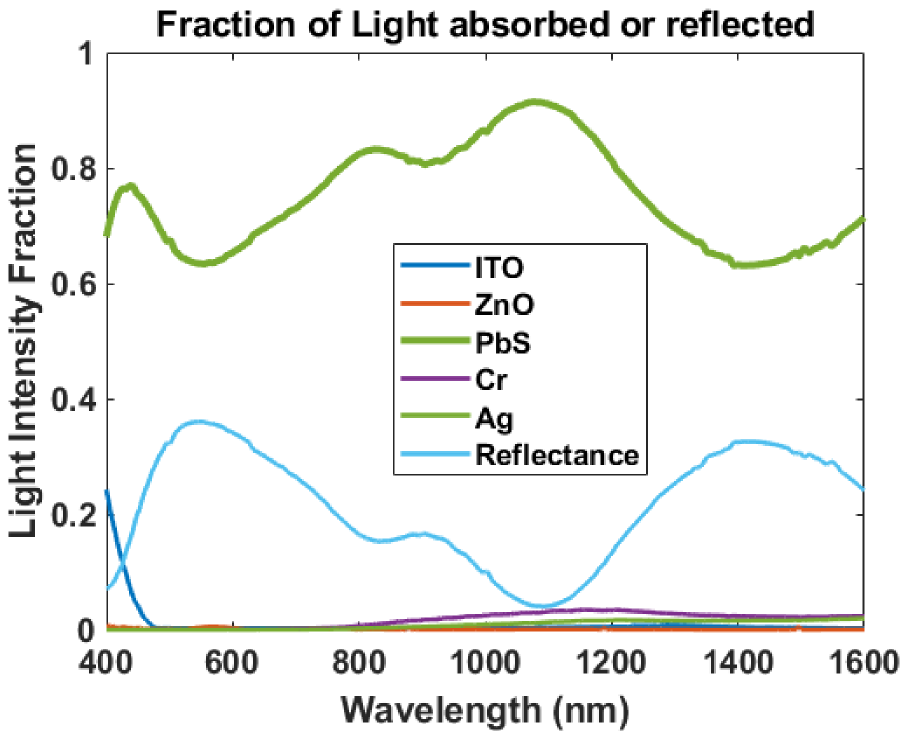

Figure S1. Simulated EQE spectra for our device.

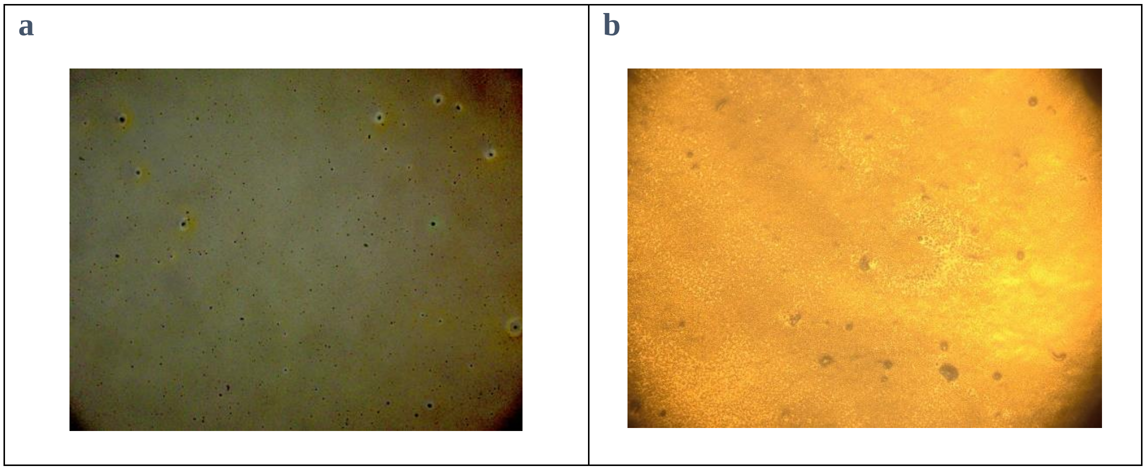

Figure S2. a. One layer of spin-coated film surface has many pinholes b. Four layers of spin-coated film surface has no pinhole.

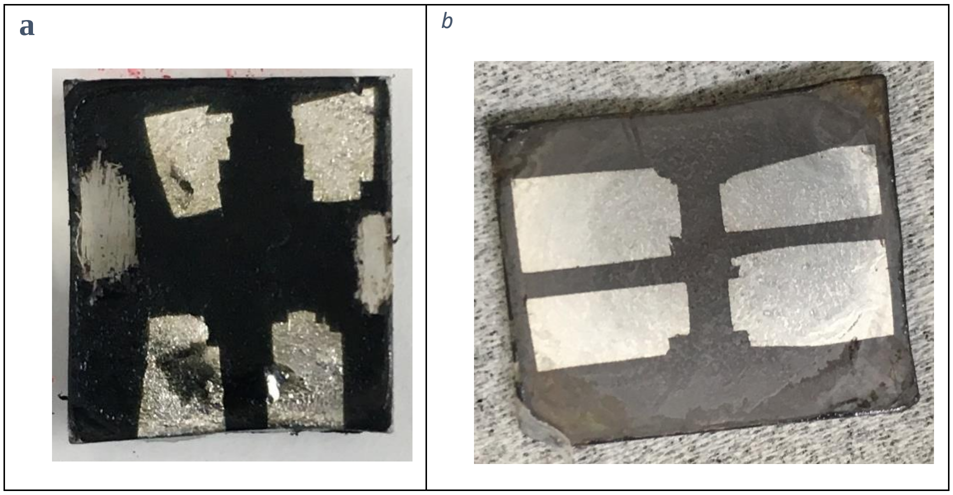

Figure S3. a. Ag electrode causes crack on the surface b. Cr-Ag electrode causes no crack on the surface after five days.
